# Improving Anticancer Therapy with Naringenin-Loaded Silk Fibroin Nanoparticles

**DOI:** 10.3390/nano10040718

**Published:** 2020-04-10

**Authors:** Marta G. Fuster, Guzmán Carissimi, Mercedes G. Montalbán, Gloria Víllora

**Affiliations:** Department of Chemical Engineering, Faculty of Chemistry, University of Murcia (UMU), Campus de Espinardo, 30100 Murcia, Spain; marta.g.f@um.es (M.G.F.); guzmanaugusto.carissimin@um.es (G.C.); gvillora@um.es (G.V.)

**Keywords:** anticancer activity, naringenin, silk fibroin, nanoparticle, green synthesis, ionic liquids, cytotoxicity

## Abstract

Naringenin (NAR), a flavonoid present in a variety of fruits, vegetables and herbs, exhibits a wide range of pharmacological effects, including anticancer activity. Nevertheless, its application in cancer therapy is limited due to its low bioavailability at the tumour site because of its poor solubility in water and slow dissolution rate. To improve the therapeutic efficacy of NAR, emergent research is looking into using nanocarriers. Silk fibroin (SF), from the *Bombyx mori* silkworm, is a biocompatible and biodegradable polymer with excellent mechanical properties and an amphiphilic chemistry that make it a promising candidate as a controlled release drug system. The aim of this work is to synthesize naringenin-loaded silk fibroin nanoparticles (NAR-SFNs) by dissolving the SF in the ionic liquid 1-ethyl-3-methylimidazolium acetate, using high-power ultrasounds and rapid desolvation in methanol followed by the adsorption of NAR. The NAR-SFNs were characterized by dynamic light scattering, Fourier transform infrared spectroscopy and thermogravimetric analysis. The drug loading content and encapsulation efficiency were calculated. The drug release profile best fitted a first order equation. The cytotoxicity effects of free NAR, bare silk fibroin nanoparticles (SFNs) and NAR-SFNs were assessed on HeLa and EA.hy926 cells via 3-(4,5-dimethylthiazol-2-yl)-2,5-diphenyltetrazolium bromide (MTT) assay. The results demonstrated the higher in vitro anticancer potential of synthesized NAR-SFNs than that of free NAR in HeLa cancer cells.

## 1. Introduction

Naringenin (5,7-dihydroxy-2-(4-hydroxyphenyl)chroman-4-one, NAR) is a natural aglycone flavonoid and metabolite of naringin. It is widely distributed in a variety of fruits, vegetables and herbs. As a flavonoid compound, its beneficial pharmacological effects on biological systems as an anticancer, antioxidant, anti-inflammatory, hepatoprotective and anti-mutagenic compound have been described [1]. Among these, the antioxidant abilities of NAR have been attributed to its chemical structure, in which the three hydroxyl groups in its aromatic rings can donate hydrogen to reactive oxygen species (ROS) [2]. ROS are highly reactive compounds and products of oxygen metabolism in cells. Cancer cells exhibit high levels of ROS, which help to propagate mutation through the oxidation of DNA, and also have many other damaging effects [3]. Unfortunately, as mentioned above, their poor water solubility (<0.01 mg/mL), low permeability, instability and propensity to gastrointestinal degradation hinder their ability to reach the systemic circulation, thus limiting any in vivo biological effects. Therefore, NAR consumed in food or administrated intravenously is incapable of providing prolonged concentration levels that are high enough to provide beneficial activities. Hsiu et al. [4] found that when NAR was orally administrated to rabbits the absolute bioavailability achieved was only 4%, considerably lower than the 80% achieved by common anti-inflammatory drugs such as ibuprofen and aspirin. In attempts to overcome these problems and to allow the clinical applications of this compound in humans, the use of novel drug delivery systems (NDDS) has shown great promise [5,6,7]. 

In order to improve the therapeutic efficiency and bioavailability of drugs, scientists have developed a great variety of NDDS. Among these, synthetic or natural nanocarriers, such as solid lipid nanoparticles [5], polymer-nanoparticles [6] and inorganic nanoparticles [7], have been studied. The use of nanoparticles has increased in recent years, due to their unique properties, including their small size and the possibility of targeting cells or tissues, which makes them promising delivery systems for increasing the bioavailability, efficacy and solubility of drugs [8,9,10]. The physical characterization of nanoparticles is a key factor for evaluating their potential for applications for which they have been designed because it is not only their chemical composition that affects their ability to act as successful nanocarriers but also their structure, size distribution and electrostatic properties.

As regards NAR, many approaches have been tried, including the use of β-cyclodextrin inclusion complexes [11], phospholipid complexes [12] and polymeric nanoparticles [10]. Of these, one of the most promising delivery systems are nanoparticles (particles with a diameter of 10–200 nm), which can act as drug-loaded particles composed of natural or synthetic polymers. Moreover, recent research has tended to focus on biopolymers to encapsulate NAR and other drugs since they are biodegradable, natural and environmentally friendly [8]. In this respect, silk fibroin (SF) is a protein and natural polymer found in *Bombyx mori* cocoons. Its use as biomaterial and nanocarrier is of great interest due to its relatively inexpensive nature, wide availability and inherent properties, including biocompatibility, environmental stability, non-toxicity and a controllable rate of biodegradation [13].

Silk is produced by a variety of insects and arachnids. However, only the silk from silkworms has historically been produced on an industrial scale and recently it has been used as biomaterial, due its long-lasting properties and abundance [14]. Silk from the *Bombyx mori* silkworm is composed of two proteins: fibroin and sericin, the former found in the interior of the thread, where it constitutes about 70% of the weight of the silk, while the latter represents the remaining 30% and is found in the thin layer surrounding the interior thread [15]. Structurally, SF is a copolymer formed by hydrophobic and hydrophilic blocks, with a primary structure composed mostly of the glycine, alanine (both hydrophobic) and serine (hydrophilic) amino acids. The hexapeptide sequence Gly-Ser-Gly-Ala-Gly-Ala allows the formation of compact blocks of anti-parallel stacked strands joined by hydrogen bonds and hydrophobic interactions, known as β-sheets [16]. As a protein, SF is susceptible to biological degradation by proteolytic enzymes such as chymotrypsin, actinase and carboxylase, which bind to and digest the SF. However, the rate of biodegradation can be controlled and delayed by altering the degree of crystallinity (β-sheet) during the preparation of the SF [17]. 

It has been demonstrated that the side chains of the amino acidic residues in SF can interact with small molecules, adsorbing them onto their surface, a result of which is that the stability of the molecules may increase [18]. Owing to its structure and amino acid composition, SF is able to attract and adsorb other molecules through hydrogen bonding and hydrophobic/hydrophilic interactions. As with any other molecule, natural electrostatic (charge-charge) forces, including van der Waals forces, are also at play [19]. Successful adsorption results in an external coating of molecule(s) around the silk nanoparticle surface. The possibility of adsorbing both drug and cell-targeting protein receptors, thus ensuring that the drug is delivered to the desired cells, is also highly promising. For this reason, adsorption is considered one of the best silk-based immobilization strategies for improving the stability and bioavailability of compounds [8]. In comparison to encapsulation, the adsorption technique has the advantage of being mild, straightforward and cheap (less equipment and fewer materials are required). However, the extent to which drugs can be loaded is entirely dependent on the complex nature of the interactions between the SF and the drug, so that each particular case must be examined.

Many studies have confirmed the potential of silk fibroin nanoparticles (SFNs) for capturing and releasing herbal and model drugs [8,9,20,21]. The study presented here consists of a straightforward method which involves mixing the drug with the pre-formed SFNs to see whether the two can physically adsorb to one another through natural intermolecular interactions. According to the literature, this has not been attempted previously with NAR. Furthermore, SFNs have traditionally been synthesized using two solvent systems to dissolve SF fibres: an ionic aqueous solution of, for example, 50 wt. % CaCl_2_ or 9.3 M LiBr [22], or an ionic hydro-alcoholic solution, such as a CaCl_2_/ethanol/water (Ajisawa’s reagent) [23], which require dialysis in deionised water to remove salts. However, the resulting solutions are unstable. As an alternative, it is possible to use an inefficient process that involves a toxic organic solvent such as hexafluoroisopropanol (HFIP) but also requires the concentration of dialyzed aqueous fibroin solution and lyophilization [24]. The process described herein, which was developed recently by our research group, uses solely high-power ultrasounds and the ionic liquid 1-ethyl-3-methyl imidazolium acetate ([emim^+^][acetate^−^]) for the silk dissolution. Previously, the SF was dissolved in other ionic liquids (ILs) such as 1-butyl-3-methylimidazolium chloride ([bmim^+^][Cl^−^]) to form a stable SF solution [25]. The solubility of SF in ILs is attributed to the ability of the anion (mainly halogens or small carboxylates) to disrupt the hydrogen bonds in the SF β-sheets [26]. The main advantage of using ILs as the solvent in this process is that the total number of steps is reduced, as the cocoon can be dissolved directly in the selected IL as a function of its properties [27]. ILs are also a considerably greener option compared with using HFIP, which is a volatile, corrosive and toxic solvent. The new method is faster, more efficient and considerably greener [27], as ILs have negligible vapour pressure and can be reused, which has added further interest to SFNs and their potential use as nanocarriers. 

The main aim of the present study is two-fold: (i) to produce NAR-SFNs using this new process in order to evaluate whether the SFNs and NAR can adsorb to one another and form a stable and functional NDDS and (ii) to evaluate the cytotoxic activity of free NAR, SFNs and NAR-SFNs in HeLa and EA.hy926 cells. The first objective was achieved by measuring the concentration of NAR during the loading stage, thus permitting the drug loading content (DLC) and encapsulation efficiency (EE) to be assessed. Drug release was measured in phosphate-buffered solution (PBS) in physiological conditions. In addition, the SFNs and NAR-SFNs prepared were characterized to assess their physical properties (hydrodynamic diameter, zeta potential, and secondary structure) using dynamic light scattering (DLS) and Fourier transform infrared spectroscopy (FTIR) and thermal degradation using thermogravimetric analysis (TGA).

## 2. Materials and Methods 

### 2.1. Materials

Silk of Bombyx Mori was obtained from the cocoons of silkworms reared in the sericulture facilities of IMIDA (Murcia, Spain) and raised on a diet of fresh Morus alba L. leaves. This silk was treated to remove the sericin with an aqueous solution of Na_2_CO_3_ (0.05N), boiling for 45 min. After washing with distilled water and air-drying, the resulting fibers have a bright white cotton-like appearance. The SF was dissolved in [emim^+^][acetate^−^] by high-power ultrasounds, as previously reported [27]. The ionic liquid (97% purity) was purchased from IoLiTec GmbH (Frankfurt, Germany) and was used without further purification. NAR (95% purity) was provided by Sigma-Aldrich (Madrid, Spain). Purified water (18.2 MΩ·cm at 25 °C; from a Millipore Direct-Q1 ultrapure water system, Billerica, MA, USA) was used throughout. All other chemicals and solvents were of analytical grade and were used without further purification.

### 2.2. Synthesis of Silk Fibroin Nanoparticles (SFNs) 

**Scheme 1:**
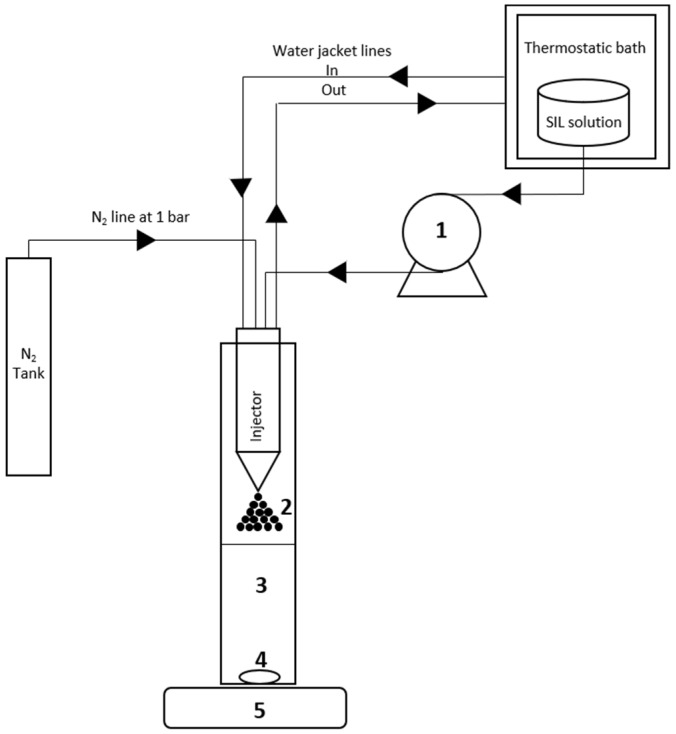
Experimental apparatus used for the silk fibroin nanoparticle (SFN) synthesis process. (**1**) peristaltic pump; (**2**) SF-[emim^+^][acetate^−^] aerosol generated with pressurized N_2_; (**3**) Methanol at −20 °C; (**4**) stirring bar; (**5**) stirring plate.

The process used to synthesize SFNs was based on the technique previously described by Lozano-Pérez et al. [27] with modifications. Briefly, an SF-[emim^+^][acetate^−^] (SIL) solution (10 wt. %) was prepared by adding 0.5 g of SF to 4.5 g of [emim^+^][acetate^−^] and dissolved with the aid of the ultrasonicator. A program of alternating pulses of 30 s was used for a time of approximately 20 min, with an amplitude of 30% and maximum temperature of 75–80 °C to prevent degradation of the protein (measured with the temperature probe that incorporates the equipment). The SF was added in small portions, checking each time that it was completely dissolved. Using the vortex mixer, 3 mL of ultrapure water was slowly added to this solution (freshly prepared or kept at 40 °C in the oven beforehand) to reduce viscosity, reaching a final SF concentration of 6.66 wt. % in the SIL solution. The sample was placed in a thermostatic bath at a temperature of 60 °C and then pumped and precipitated through a thermostatically controlled 0.7 mm two-fluid nozzle (from a Mini Spray Dryer B-290, BÜCHI Labortechnik, Flawil, Switzerland, Part No. 044698) into 100 mL of methanol (−20 °C) while stirring (1800–2200 rpm). The feed rate of the SIL solution into the spray was maintained at 13.64 mL/min (Range 500, 12.5 rpm). The nitrogen pressure was maintained at 1 bar throughout the process. Once the precipitation process was completed, the precipitated solution was stirred for two hours. Scheme 1 shows the experimental apparatus used for the synthesis of the SFNs. After the synthesis process, particles were thoroughly washed with methanol and water to remove the ionic liquid by centrifugation at 13,400 rpm. After four washes (two with methanol and two with water), the particles were resuspended in Milli-Q water and aliquots were poured onto Ø 90 × 14 mm Petri dishes. After freezing the samples overnight at −20 °C, they were placed in the lyophilizer (Thermo Scientific, Waltham, MA, USA) for 72 h, at −55 °C and 0.5 mbar to obtain dry particles.

To recovery the [emim^+^][acetate^−^] from the methanolic fractions, a BÜCHI RE-111 rotary evaporator, at 80 °C and 80 mbar, was used. As mentioned above, due to the very low vapour pressure of the IL, it can be separated from the methanol and both later reused. The recycled IL was filtered through 0.22 µm pore diameter, then maintained for 24 h in the oven at 100 °C to remove water, after which it was kept in a desiccator under vacuum and phosphorus pentoxide atmosphere until reuse.

### 2.3. Preparation of Naringenin-Loaded Silk Fibroin Nanoparticles (NAR-SFNs)

Three different NAR/SFN ratios (w:w) of the loading solutions were tested in this study, each performed at least in duplicate being 1:1, 1:2 and 1:4. The corresponding concentrations of NAR in the ethanolic loading solutions were 5, 2.5 and 1.25 mg/mL, respectively. The amount of SFNs was 200 mg in all the experiments. In each case, the NAR and SFNs were brought into contact in a 50 mL Falcon^®^ tube and subjected to ultrasonication for 5 min using 30% amplitude with pulses of 15 s ON and 15 s OFF and then gently stirred at 30 rpm in a MX-RD-ProAnalog Tube Rotator (Scilogex, Rocky Hill, CT, USA), at 4 °C in the dark for 24 h to ensure thorough mixing and adsorption of the drug to the SFNs. Following this, the tubes were centrifuged for 15 min and the supernatants were removed and analysed by high-performance liquid chromatography (HPLC). The nanoparticles were then washed with ultrapure water to remove the remaining ethanol. The amount of drug loaded onto the SFNs was determined by an indirect method, measuring the concentration of NAR in the supernatant after centrifugation and in the initial ethanolic solution and calculating the quantity (*w*) of NAR loaded onto the particles, as shown in Equation (1).

(1) NAR Loaded on SFNs (w)=NAR in Loading Solution (w)−NAR in Supernatant (w)

### 2.4. Determination of Naringenin (NAR)

High-performance liquid chromatography (HPLC) was used to measure the NAR concentration in the NAR/ethanol drug-loading solutions. The HPLC equipment (Waters Alliance system from Waters, Milford, MA, USA) was equipped with an e2695 separation module, an injector valve with a 100 µL loop, and a Waters 2998 photodiode array (PDA) detector. The results were processed by Empower 3, Waters software (Waters Corporation, Milford, MA, USA). Separations were performed on a Sunfire^TM^ analytical column (C18, 5 μm, Ø 4.8 × 150 mm). The mobile phase consisted of a mixture 50% methanol (A) and 50% water (B). The PAD was set at 200–400 nm wavelength and the chromatogram was detected at 280 nm. The analysis was performed at 35 °C (column oven temperature), with a 1mL·min^−1^ flow rate and the injection volume was 2 μL. The duration of each measurement was 11 min and the NAR peak appeared around 8.1 min. The assay was linear over the range 0.05–0.045 mM with the standard regression equation of the peak area [×10^6^ uV·s] of NAR (*Y*) to NAR concentration (*X*) being as follows: *Y* = −61.674*X* (correlation coefficient *R^2^* = 0.9998).

The drug content in PBS (PBS 1x; pH 7.4, with 0.5% v/v Tween 80) solutions was measured by Ultraviolet-Visible (UV-Vis) spectroscopy. Analyses were carried out using a UV-Vis HELIOSα spectrophotometer (Thermo Scientific, Waltham, MA, USA) and good linear correlations were obtained between absorbance (*Y*) and NAR concentration (*X*) in the range 0.005–0.043 mM (*Y* = 16.232*X*), with a correlation coefficient of *R^2^* = 0.9983. The spectrophotometric detection was determined at an absorption maximum of 290 nm.

### 2.5. Stability of NAR in Ethanol

A test was carried out to investigate the stability of NAR in ethanol, using a 1 mM solution of NAR in ethanol, which was divided into two separate flasks. One was left at room temperature (about 25 °C) in natural light, while the other was refrigerated (4 °C) in darkness. The NAR concentration of both was measured daily by HPLC over 12 days.

### 2.6. Characterisation of Nanoparticles

#### 2.6.1. Dynamic Light Scattering (DLS)

After lyophilisation was completed for each batch of particles (loaded and unloaded), the mean hydrodynamic diameter (Z-average), the polydispersity index (PdI), the zeta potential and the electrophoretic mobility were measured by DLS using a Zetasizer Nano ZS instrument (Malvern Instruments Ltd., Worcestershire, UK). All measurements were obtained in ultrapure water at 25 °C and at a 173° angle relative to the source. Before the measurements, each sample was sonicated for 3 min at 30% amplitude with pulses of 15 s ON and 15 s OFF; the NAR-SFNs concentration was 0.66 mg/mL. All measurements were performed in triplicate and values expressed as mean ± SD.

#### 2.6.2. Attenuated Total Reflectance Fourier Transformed Infrared Spectroscopy (ATR-FTIR)

Infrared spectral data of the SFNs, NAR and NAR-SFNs were obtained by ATR-FTIR in a Nicolet iS5 spectrometer coupled to a diamond crystal iD7 ATR module (Thermo Fischer Scientific, Waltham, MA, USA). OMNIC Software V9.9.471 (Thermo Fischer Scientific, Waltham, MA, USA) was used for the control and processing spectral data. Interferograms were recorded at a resolution of 2 cm^−1^ in the spectral range of 1750–900 cm^−1^ with a zero-filling factor of 2 and Fourier-transformed using the Blackman-Harris 3-term apodization function. Each measured spectrum was averaged from 64 scans. A background spectrum without sample with the same number of scans was collected before each measurement.

Spectra of the samples were acquired by placing 2.5 µL of the nanoparticle dispersion or NAR solution (15 mg/mL) on the top of the ATR-crystal after drying under a gentle stream of nitrogen.

#### 2.6.3. Field Emission Scanning Electron Microscopy (FESEM)

FESEM was used for the morphological characterization of NAR-SFNs, using a FEI Scios^TM^ microscope (Thermo Scientific, Waltham, MA, USA). The NAR-SFNs sample was deposited as a powder and coated with a thin gold layer.

#### 2.6.4. Thermogravimetric Analysis (TGA)

The thermal behaviour of NAR-SFNs was determined by TGA and compared with the thermal profiles of the SFNs and NAR individually. The measurements were carried out by a thermal gravimetric analyser (TA instruments, SDT 2960, Waters LLC, New Castle, DE, USA), in a temperature range of 25−800 °C and heating rate of 10 °C/min under an inert nitrogen dynamic atmosphere. Weight loss was recorded and plotted against temperature. The derivative of the weight loss curves (DTGA) was calculated and also plotted versus temperature.

#### 2.6.5. Drug Loading Content (DLC) and Entrapment Efficiency (EE) of NAR-SFNs

DLC and EE quantify the success of the loading with respect to the mass of the nanoparticles and the mass of the drug, respectively. These were calculated according to Equations (2) and (3):

(2) $$DLC\;\left(\%\right)=\frac{Mass\;of\;NAR\;Loaded\;onto\;SFNs}{Mass\;of\;NAR-SFNs}\times 100$$

(3) $$EE\;\left(\%\right)=\frac{Mass\;of\;NAR\;Loaded\;onto\;SFNs}{Mass\;of\;NAR\;added\;to\;SFNs}\times 100$$

where the mass of NAR-SFNs is the mass of drug-loaded SFNs obtained at the final of the synthesis process.

#### 2.6.6. In Vitro Cytotoxicity 

Human cervical cancer cells (HeLa) and Human umbilical immortalized cells (EA.hy926) were purchased from the American Type Culture Collection (ATCC, USA). The culture medium used was Dulbecco’s Modified Eagle Medium (DMEM) with a low content of glucose (1 g/L). The medium was supplemented with 10% (v/v) fetal bovine serum (FBS), 1 mM glutamax, 1% antibiotics (penicillin-streptomycin) and 1 mM pyruvate at 37 °C under a humidified atmosphere containing 5% CO_2_ for both cell lines. Cells were subcultured using a solution of 0.25% trypsine-0.25 mM ethylenediaminetetraacetic acid (EDTA) and the medium was changed twice a week. It was checked that all cell lines were mycoplasma-free, before and after the experiments.

A total of 5 × 10^3^ cells/well were seeded onto a 96-well plate and incubated at 37 °C. After 24 h, the culture medium of each well was replaced with fresh medium and cells were treated with different concentrations of free NAR (0.39–50 µg/mL), SFNs (1.95–250 µg/mL) and NAR-SFNs (1.95–250 µg/mL). In each experiment, growth medium without nanoparticles was used as a control. Cells were incubated at 37 °C for 48 h. Then, the media was removed and 200 µL of MTT (3-(4,5-dimethylthiazol-2-yl)-2,5-diphenyltetrazolium bromide) solution at a final concentration of 1nmg/mL were added and left in darkness for 4 h, after which the MTT was removed and 100 µL of dimethyl sulfoxide (DMSO) were added. Absorbance was measured in a microplate reader (Floustar Omega) spectrophotometer at 560 nm. Each sample was tested in three independent sets with triplicate points.

#### 2.6.7. Procedure for Determining Free Radical Scavenging Activity of NAR-SFNs by DPPH Assay

As stated above, NAR is widely considered as an antioxidant compound with activity against oxidative damage of human cells. The ability of the NAR-SFNs to annihilate the DPPH radical (2,2-diphenyl-1-picrylhydrazyl) was investigated using the method described by Blois [28]. Compounds that show radical scavenging activity, like NAR, induce colour bleaching of a DPPH·solution, which can be measured by UV-Vis spectrophotometry [29]. Aliquots of 100 μL of the 1 mM DPPH·stock solution in methanol, recently prepared and protected from light, were added to the Eppendorf^®^ vials containing a mixture of 100 μL of water and 800 μL of methanol and 10.83 mg of NAR-SFNs. The reaction mixture was incubated for 30 min at 25 °C in darkness and the absorbance was recorded at 517 nm using a UV-Vis HELIOSα spectrophotometer (Thermo Scientific, Waltham, MA, USA). As standard control, ascorbic acid solutions (0–2.5 nmol/mL in methanol) were used. The radical scavenging activity is presented as Ascorbic Acid Equivalents per milligram of NAR in the NAR-SFNs (nmol AAE/mg NAR). The ability to scavenge the DPPH radical was also expressed in terms of the inhibition percentage calculated with Equation (4):

(4) $$Inhibition\;\left(\%\right)=\frac{A_{0}-A_{1}}{A_{0}}\times 100$$

where *A_0_* is the absorbance of the control and *A_1_* is the absorbance of the sample of NAR-SFNs. Apart from for the sample of NAR-SFNs, inhibition percentage of the ascorbic acid solutions was also calculated from their respective absorbances and represented versus their concentrations.

The experiment was repeated three times and the results are expressed as mean ± standard deviation (SD). 

#### 2.6.8. Naringenin Release from NAR-SFNs

In order to study the drug release properties of the NAR-SFNs, drug release experiments were carried out with the three NAR-SFNs (obtained with different NAR/SFN loading solution ratios) in PBS 1x (pH = 7.4) with 0.5% of TWEEN 80. The concentration and pH of this medium is similar to those of blood. Thirty milligrams of NAR-SFNs were dispersed in 1 mL of PBS 1x by ultrasonication and incubated at 37 °C in a tube shaker for 2 days. At predetermined time points (0.5, 1, 1.5, 2, 2.5, 3, 3.5, 4, 5 and 24 h), the samples were centrifuged for 15 min at 13400 rpm. The concentration of NAR in the supernatant was measured by UV-Vis spectrophotometry as described in Section 2.4. One millilitre of fresh PBS 1x (pH = 7.4) was added to the Eppendorf vial, which was placed again in the shaker. These experiments were carried out in triplicate for every sample, and experimental data were fitted using four release kinetic models found in the literature (Zero order, First order, Ritger-Peppas and Higuchi) [30] in order to elucidate the release mechanism of NAR from the NAR-SFNs in PBS.

### 2.7. Statistical Analysis

Data were presented as mean ± SD (standard deviation), calculated from three independent samples per condition by using GraphPad Prism 8.0.1 software (GraphPad Software, San Diego, CA, USA). As normality (Kolmogorov-Smirnov, *p* > 0.05) and homoscedasticity (Levene, *p* > 0.05) were met, the statistical significance was determined using the parametric tests of Tukey (*p* < 0.05) and ANOVA (*p* < 0.05) for the comparisons of two or more groups, respectively.

## 3. Results and Discussion

### 3.1. Stability of NAR in Ethanol

The stability of NAR/ethanol solutions under the two sets of experimental conditions was studied: natural light at room temperature (about 25 °C), and darkness in a refrigerator (4 °C). Over the course of 12 days, the concentration of the NAR/ethanol solution exposed to light and room temperature decreased from 0.279 to 0.235 g/L, while the concentration of the solution kept in the fridge decreased from 0.279 to 0.271 g/L. Although the decrease in NAR concentration was small in both cases, all the solutions containing NAR or NAR-SFNs were kept refrigerated throughout the study and were never exposed to light or ambient room temperature for more than one hour.

### 3.2. Characterization of SFNs and NAR-SFNs

#### 3.2.1. Dynamic Light Scattering (DLS)

**Figure 1:**
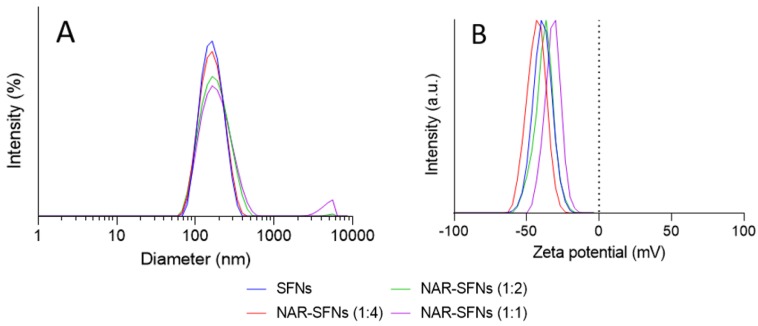
Size distribution (**A**) and zeta potential (**B**) of virgin silk fibroin nanoparticles (SFNs) (blue) and naringenin-loaded silk fibroin nanoparticles (NAR-SFNs) prepared from the three NAR/SFN ratios: 1:4 (red), 1:2 (green) and 1:1 (purple).

The SFNs and NAR-SFNs (1:4, 1:2, 1:1) were characterized by DLS to ascertain their hydrodynamic diameter (expressed as Z-average), zeta potential, PdI and electrophoretic mobility. As can be seen in Table 1, there was a clear correlation between drug concentration and particle size: as the drug concentration increased, so did the particle size. By contrast, the absolute values of the zeta potential were seen to gradually decrease. The PdI gradually increased with the NAR concentration in the loading solution, indicating that the overall range of particle size increased. This suggests that the higher the drug concentration (and hence DLC values), the greater the variation in the proportionality of loading between particles. At 5 mg/mL of loading solution (NAR-SFNs 1:1), the PdI reached 0.22, indicating that there was more than one size distribution, i.e., other particle(s) of a distinct size existed within the sample, as is shown in Figure 1. This was probably due to the fact that a considerably high concentration of free NAR particles was not loaded, suggesting that at this concentration the SFNs begin to become fully saturated.

In summary, the NAR-SFNs increased in size with the NAR concentration in the loading solution, but still retained an average particle size of <200 nm, while maintaining a relatively high absolute zeta potential value. In terms of their physical characteristics, therefore, they appear to be entirely suitable as potential nanocarriers.

#### 3.2.2. Attenuated Total Reflectance Fourier Transformed Infrared Spectroscopy (ATR-FTIR)

**Figure 2:**
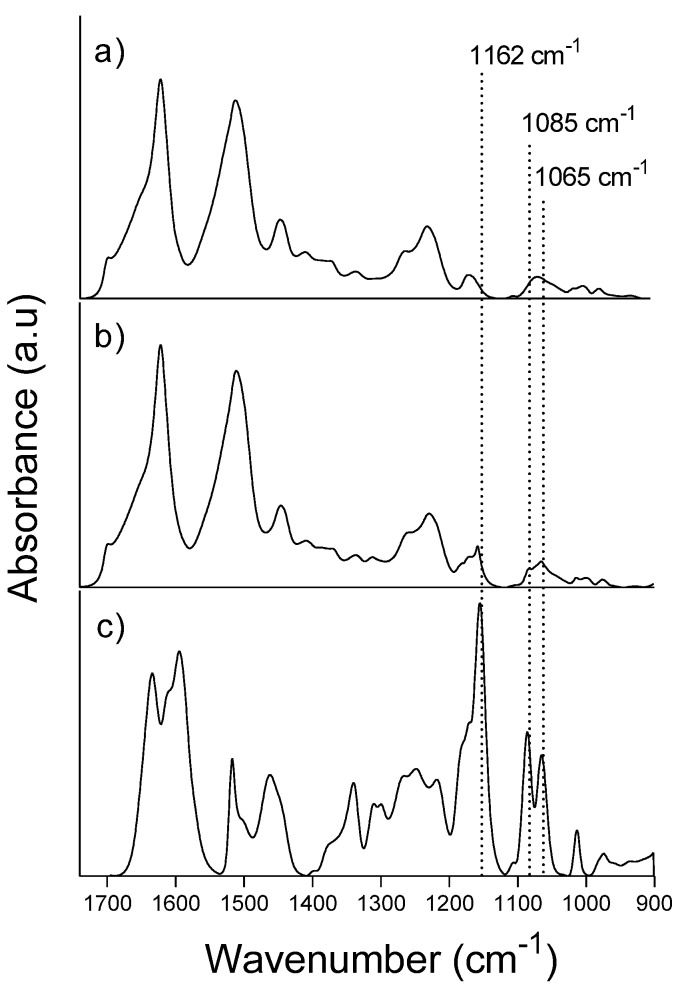
Infrared spectra of virgin SFNs (**a**), NAR-SFNs 1:1 (**b**) and NAR (**c**).

ATR-FTIR was carried out to obtain information about the secondary structure of the proteins based on the position of the signals and relative intensity. Figure 2 shows the infrared spectra of the virgin SFNs (a), NAR-SFNs 1:1 (b) and free NAR (c).

The characteristic bands of SF, as also shown by other authors [31,32,33], can be seen in the three large peaks (not highlighted) of Figure 2a,b at approximately 1620, 1515 and 1230 cm^−1^, denoting amide I, II and III, respectively [34]. Amide I, particularly, presents a band split produced by the transition dipole coupling [35] characteristic of the antiparallel β-sheet of silk II at 1700 cm^−1^ [36]. The spectrum of NAR-SFNs contains several peaks from NAR, the most noticeable at 1162, 1085 and 1065 cm^−1^, denoted by the dotted lines in Figure 2c. According to Machado et al. [37], bands at 1085 and 1065 cm^−1^ arise from aromatic skeletal vibration of the flavone ring. The band at 1162 cm^−1^ is due to HOC bending of the NAR ring with two hydroxyl groups [38].

#### 3.2.3. Field Emission Scanning Electron Microscopy (FESEM)

**Figure 3:**
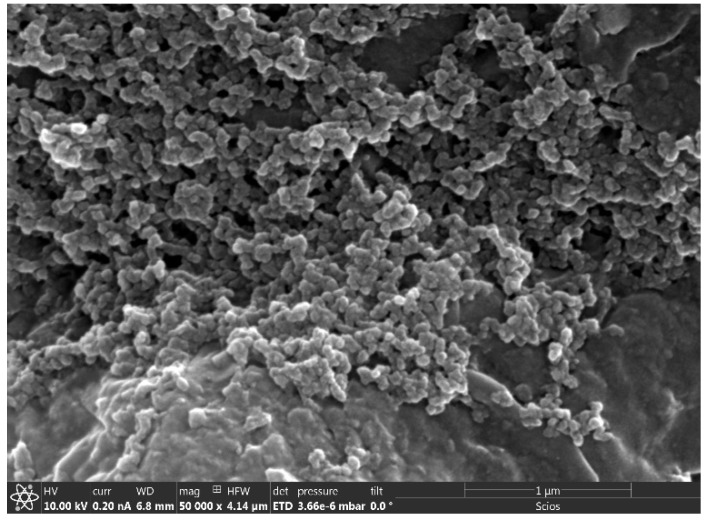
Field emission scanning electron microscopy (FESEM) picture of NAR-SFNs 1:1 (50,000×).

The morphology and size of the NAR-SFNs were examined by FESEM, as shown in Figure 3. It was found that NAR-SFNs are globular granules, as mentioned by the authors for other drug-loaded SFNs [8,39]. Homogeneous size distribution can be observed, and the diameters, which are the real particle diameters, seem to be smaller than those obtained by DLS. This is probably due to the particles swelling in the water solution. DLS measurements were taken with NAR-SFNs/water suspensions, while the FESEM sample was dried. These results agree with those obtained by the authors with curcumin-loaded SFNs [8]. In addition, DLS shows the diameter including both particles and the diffusion layer, and that is another reason why the size of DLS is larger than that from FESEM images.

#### 3.2.4. Thermogravimetrical Analysis (TGA)

**Figure 4:**
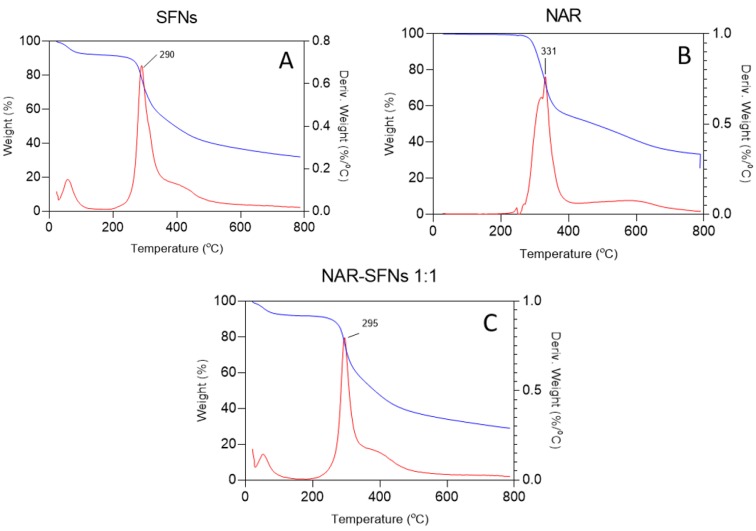
Thermogravimetrical analysis (TGA) and first derivative of the TGA (DTGA) of the (**A**) SFNs, (**B**) NAR and (**C**) NAR-SFNs 1:1.

TGA analysis were carried out on samples of pure NAR as well as on drug-free and drug-loaded nanoparticles. The results obtained for the TGA of SFNs are depicted in Figure 4a, where the thermal decomposition rate is denoted by the peak of the first derivate which corresponds to an endothermic transition (negative peak of the TGA curve). The mass loss of SFNs and NAR-SFNs in the first transition at *T* < 100 °C were due to water evaporation. As can be seen, in all cases, the weight residue percentage falls sharply after 289–331 °C, due to the degradation of SF and NAR. In the case of both types of nanoparticle, free and loaded, the reduction can be attributed to thermal degradation of β-sheet ordered structures [25,32].

#### 3.2.5. Drug Loading

Table 2 summarizes the values of DLC and EE calculated for the three types of NAR-SFNs obtained from Equations (2) and (3), respectively. It can be seen that the DLC increased as the NAR/SFNs mass ratio increased in the loading solution, i.e., sample 1:1 had the highest values. However, the EE maintained approximately the same value of around 20%.

#### 3.2.6. In Vitro Cytotoxicity Studies 

**Figure 5:**
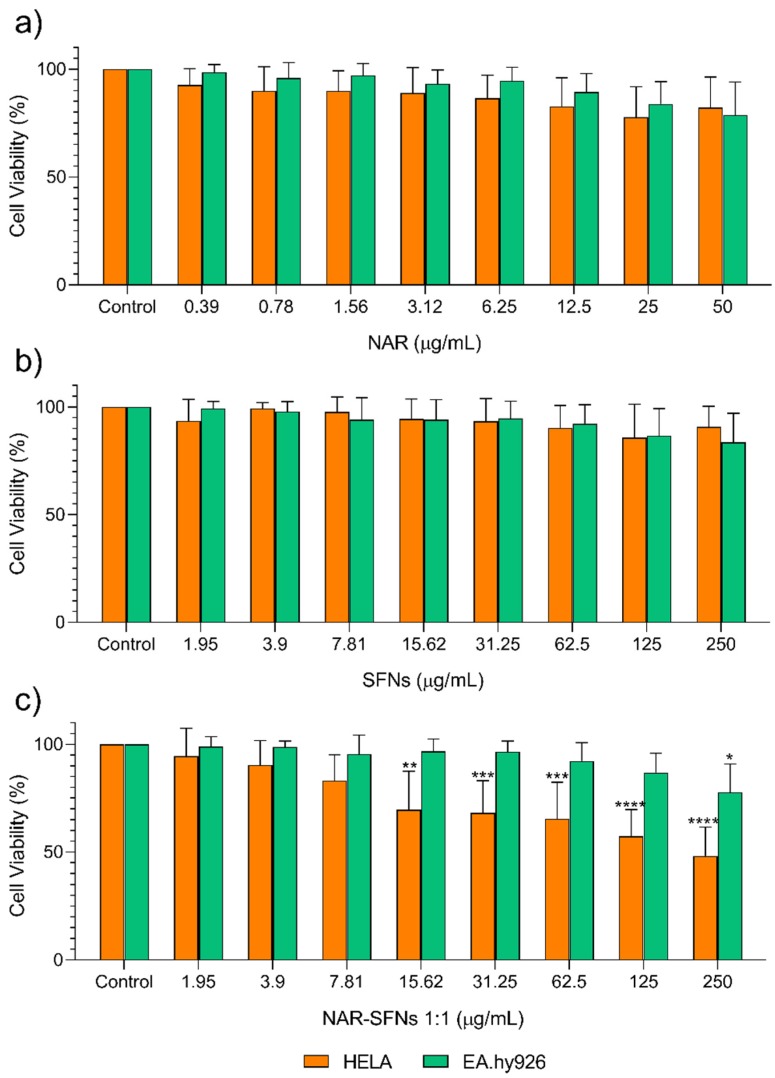
Cytotoxicity effect of free NAR (**a**), SFNs (**b**) and NAR-SFNs 1:1 (**c**) on HeLa and EA.hy926 cell lines. Data are expressed as percentage of cell viability ± SD versus concentration. * indicates *p* < 0.05, ** indicates *p* < 0.01, *** indicates *p* < 0.001 and **** indicates *p* < 0.0001, compared to control.

The in vitro cytotoxic effects of free NAR, SFNs and NAR-SFNs were assessed with the MTT assay after 48 h of exposure in two different cell lines: HeLa and EA.hy926. In this study, the cell lines were selected due to their features and origin in order to determine if the synthesized NAR-SFNs have anticancer activity. HeLa and EA.hy926 are both human cell lines. HeLa cells are derived from cervical carcinoma and have been widely used in cytotoxicity studies and EA.hy926 cells are healthy cells which coat the inside of blood vessel and are one of the most used and best characterized human vascular endothelial cell lines [40]. To study the effects of free NAR, it was evaluated at concentrations between 0.39 and 50 µg/mL. The DLC data of NAR-SFNs 1:1, which are collated in Table 2, were taken into account to test loaded nanoparticles and the concentration range 1.95–250 µg/mL was assessed in wells, using the same concentration of NAR in the SFN-NARs as in the experiments with free NAR. The same conditions were used for SFN and SFN-NAR assays in order to evaluate whether cell viability decreased due to the effect of SF or NAR. After 48 h of incubation with several concentrations of SFNs and free NAR, it was seen that cell viability for both cell lines slowly decreased or remained constant as the concentration of SFNs or free NAR increased (see Figure 5). However, NAR-SFNs were more cytotoxic than SFNs and NAR for the tumour cell lines (HeLa) than for the healthy cells (EA.hy926). In this case, hardly any cytotoxic effect was observed, as can be seen in Figure 5.

The non-toxicity of NAR towards healthy cells has been demonstrated previously in several works. For example, Md et al. [41] found that solutions with different concentration of NAR did not significantly reduce the viability of SH-SY5Y cells when compared to a control (100 ± 6.0%; *p* > 0.05), while nanoemulsions of NAR significantly increased the cell viability to 112 ± 9.0% in SH-SY5Y cells. This indicated that the prepared nanoemulsions had a better protective effect against β-Amyloid-induced toxicity than free NAR. Stompor et al. [42] demonstrated that NAR had very high selectivity towards glioblastoma cells and was more than six times more toxic towards cancer cells (gliomas are one of the most aggressive and treatment-resistant types of human brain cancer) than healthy cells.

#### 3.2.7. Antioxidant Activity of NAR-SFNs

**Figure 6:**
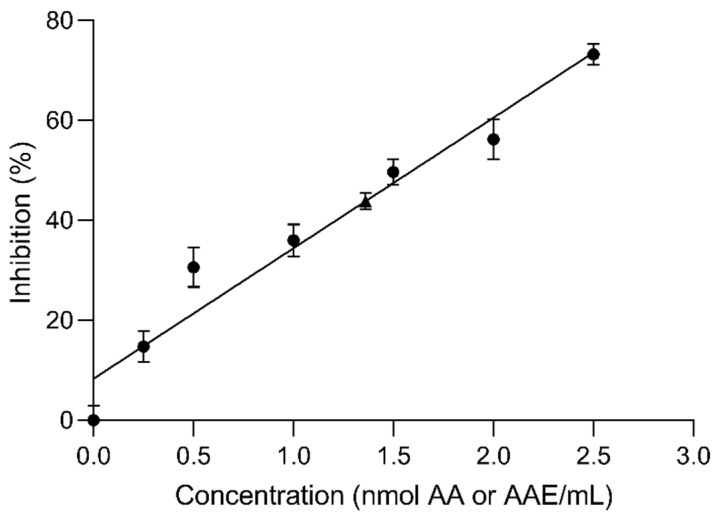
Percentage inhibition of the ascorbic acid solutions (0–2.5 nmol/mL in methanol) used as control (circles) and of the NAR-SFNs 1:1 (triangle) *vs* concentration in nmol AA/mL or nmol AAE/mL, respectively.

Flavonoids such as NAR, possess potent antioxidant activity and are strong scavengers of free radicals. In order to evaluate the effectiveness of NAR-SFNs as antioxidant, we used the DPPH scavenging assay. The DPPH assay has been previously used to test the free radical scavenging capacity of NAR [43,44]. The radical scavenging activity of NAR-SFNs has been evaluated and compared to the equivalents of an antioxidant standard (ascorbic acid) needed to obtain the same absorption reduction in the same reaction conditions. The NAR-SFNs was found to be effective in scavenging the DPPH radical. The Ascorbic Acid Equivalents per milligram of NAR in the NAR-SFNs was 1.05 ± 0.02 nmol AAE/mg NAR. The percentage inhibition of the DPPH radical was 43.9 ± 0.6%. Figure 6 shows the percentage inhibition of each ascorbic acid solution used as control to determine the AAE per mg of NAR of the studied NAR-SFNs (represented as circles) and the percentage inhibition of the NAR-SFNs (represented as a triangle). Percentage inhibition of the ascorbic acid solutions was also calculated with Equation (4) and from their respective absorbances. We have checked that, for the NAR-SFNs, the value of AAE per mg of NAR calculated from Figure 6 is the same than that calculated from the ascorbic acid calibration (1.05 nmol AAE/mg NAR, approximately). The inhibition percentage found was similar to that found for quercetin-loaded SFNs in a previous work being quercetin another flavonoid with antioxidant activity [39].

#### 3.2.8. Naringenin Release

In order to study the drug released from the NAR-SFNs into the human body, release assays were carried out. Specifically, PBS 1x medium was chosen and the tests were performed at pH and temperature values similar to those found in the human body (pH 7.4 and 37 °C, respectively). A low concentration of Tween 80 (0.5% v:v) was used to enable the NAR concentration to be determined spectrophotometrically in PBS due to the low solubility of NAR in this medium.

**Figure 7:**
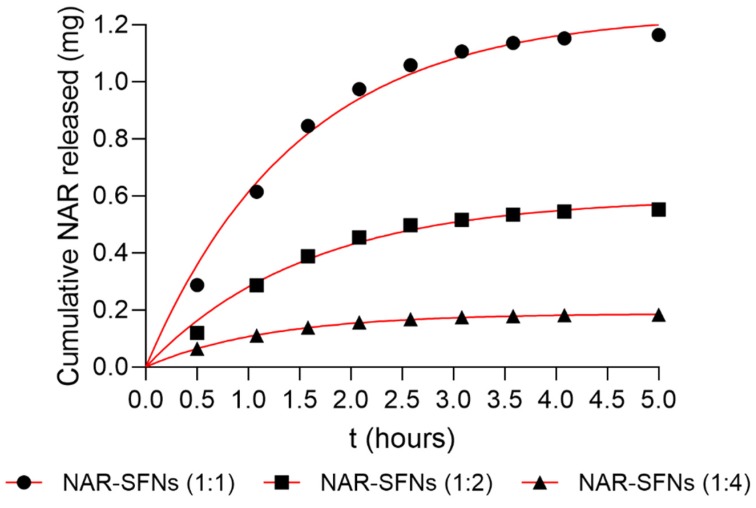
Cumulative mass of NAR released as a function of incubation time in PBS 1x (pH = 7.4) containing 0.5% of Tween 80 at 37 °C. The symbols represent experimental values. Continuous lines represent the best fit with first order equation.

Figure 7 shows the average mass of NAR released from NAR-SFNs during the release experiments at each time point. For each time, the mass of NAR was calculated as the value accumulated until this time. In the first 2 h, the initial release of nanoparticles was around 82–86% of the total drug released in all three cases. The rate of release was faster as the amount of charged drug increased, which agrees with the literature [8].

The experimental data were analysed using release models (Zero order, First order, Higuchi, Peppas), from 0.5 to 5 h, to ascertain the release kinetics of the three types of NAR-SFN. Table 3 shows the fitting equations obtained and the correlation coefficient of the fit.

Despite the complexity of the transfer processes involved in drug release, the Higuchi model provided a simple equation based on Fick’s Law, which is very easy to use. Based on a pseudo-steady-state approach, this equation establishes a direct proportionality between the amount of drug released and the square root of time. However, transport from swelling systems can often lead to release through a mechanism that does not match Higuchi or Fickian behaviour but follows an anomalous (non-Fickian) transport mechanism [45], and a more generic equation is required [30]. It was found that the NAR release profiles of NAR-SFNs made with different loading solutions best fit a first order equation. The Ritger–Peppas equation [46] has also been used to elucidate release mechanisms, since *n* in the equation is the diffusional exponent which is indicative of the transport mechanism. These authors claimed that the mechanistic limits of the diffusional exponent, *n*, are dependent on the geometry (sphere, cylinder, film) of the release device. For NAR-SFNs, the exponent *n* had a value that ranged between 0.373 ± 0.051 and 0.469 ± 0.076, indicating that NAR release from NAR-SFNs (assuming spheres) is controlled by an anomalous transport, not a pure diffusional (Fickian) mechanism.

## 4. Conclusions

NAR-SFNs were successfully synthesized by dissolving SF in the ionic liquid [emim^+^][acetate^−^] using high power ultrasounds and via atomization in methanol, followed by the adsorption of NAR during incubation. The DLC increased with increasing mass ratios of NAR/SFN during incubation in the range studied. As the DLC increased, the hydrodynamic diameter increased, and the zeta potential decreased. Both factors contribute negatively to colloidal stability, but hydrodynamic diameter <180 nm and zeta potential >30(in absolute terms) make both SFNs and NAR-SFNs suitable systems for therapeutic applications. By means of FTIR, the characteristic bands of SF were observed from the three large peaks denoting amide I, II and III. The antiparallel β-sheet secondary structure of SF was regenerated upon contact with organic polar solvent. Furthermore, the TGA study revealed that the thermal decomposition of the NAR-SFNs occurs at around 295 °C. We also found that the NAR release profiles of NAR-SFNs made from different loading solutions showed good agreement with a first order equation. Finally, it was demonstrated that NAR-SFNs retain their antioxidant activity and antitumor activity towards HeLa cells, while the viability of healthy cells hardly decreases. This feature is highly promising, since it broadens the possibility of using these nanoparticles for cancer treatment, diminishing the side effects that frequently occur with currently used therapies.

## Figures and Tables

**Table 1:** Physical characterization of the silk fibroin nanoparticles (SFNs) and naringenin-loaded silk fibroin nanoparticles (NAR-SFNs) prepared from three NAR/SFN ratios (1:4, 1:2 and 1:1).

Sample	Z-Average (nm)	PdI	Zeta Potential (mV)	Electrophoretic Mobility (µm cm/Vs)
SFNs	148.4 ± 1.3 ^a^	0.11 ± 0.01 ^a^	−39.1 ± 0.7 ^a^	−3.06 ± 0.05 ^a^
NAR-SFNs 1:4	157.6 ± 0.9 ^b^	0.12 ± 0.01 ^a^	−37.9 ± 1.5 ^a^	−2.97 ± 0.12 ^a^
NAR-SFNs 1:2	164.0 ± 1.1 ^c^	0.17 ± 0.01 ^b^	−37.8 ± 1.2 ^a^	−2.96 ± 0.09 ^a^
NAR-SFNs 1:1	180.1 ± 2.6 ^d^	0.22 ± 0.01 ^c^	−30.5 ± 0.7 ^b^	−2.39 ± 0.05 ^b^

Mean Values ± SD (standard deviation) (*n* = 3). ^a,b,c,d^ Means within each column with different letters are significantly different (*p* < 0.05), Tukey’s test.

**Table 2:** Drug loading content (DLC) and entrapment efficiency (EE) of the NAR-SFNs 1:4, NAR-SFNs 1:2 and NAR-SFNs 1:1.

Parameter	NAR-SFNs 1:4	NAR-SFNs 1:2	NAR-SFNs 1:1
DLC (%)	4.18 ± 0.19 ^a^	7.62 ± 0.24 ^b^	21.82 ± 0.40 ^c^
EE (%)	17.33 ± 0.7 ^a^	19.5 ± 0.45 ^b^	21.81 ± 0.30 ^c^

Mean Values ± SD (standard deviation) (*n* = 3). ^a,b,c^ Means within each row with different letters are significantly different (*p* < 0.05), Tukey’s test.

**Table 3:** Release models of NAR-SFNs.

Model	NAR-SFNs 1:1	NAR-SFNs 1:2	NAR-SFNs 1:4
Zero Order	*y* = 0.1788*t* + 0.4581 *R^2^* = 0.7662	*y* = 0.08783*t* + 0.2027 *R^2^* = 0.7766	*y* = 0.02414*t* + 0.08717 *R^2^* = 0.7783
First Order	*y* = 1.242(1 − e^−0.681*t*^) *R^2^* = 0.9851	*y*= 0.594(1 − e^−0.639*t*^) *R^2^* = 0.9825	*y* = 0.188(1 − e^−0.842*t*^) *R^2^* = 0.9992
Higuchi	*y* = 0.574*t*^0.5^ + 0.037 *R^2^* = 0.8825	*y*= 0.281*t*^0.5^ − 0.0033 *R^2^* = 0.8906	*y* = 0.077*t*^0.5^ + 0.031 *R^2^* = 0.8919
Ritger-Peppas	*y* = 0.632*t^n^* *R^2^* = 0.8903 *n* = 0.447 ± 0.072	*y* = 0.289*t^n^* *R^2^* = 0.8935 *n* = 0.469 ± 0.076	*y* = 0.110*t^n^* *R^2^* = 0.915 *n* = 0.373 ± 0.051

## References

[1] Nguyen-Ngo C., Willcox J.C., Lappas M. (2019). Anti-diabetic, anti-inflammatory, and anti-oxidant effects of naringenin in an in vitro human model and an in vivo murine model of gestational diabetes mellitus. Mol. Nutr. Food Res..

[2] Kumar S.P., Birundha K., Kaveri K., Devi K.T.R. (2015). Antioxidant studies of chitosan nanoparticles containing naringenin and their cytotoxicity effects in lung cancer cells. Int. J. Biol. Macromol..

[3] Gao K., Henning S.M., Niu Y., Youssefian A.A., Seeram N.P., Xu A., Heber D. (2006). The citrus flavonoid naringenin stimulates DNA repair in prostate cancer cells. J. Nutr. Biochem..

[4] Hsiu S.L., Huang T.Y., Hou Y.C., Chin D.H., Chao P.D.L. (2002). Comparison of metabolic pharmacokinetics of naringin and naringenin in rabbits. Life Sci..

[5] Ji P., Yu T., Liu Y., Jiang J., Xu J., Zhao Y., Hao Y., Qiu Y., Zhao W., Wu C. (2016). Naringenin-loaded solid lipid nanoparticles: Preparation, controlled delivery, cellular uptake, and pulmonary pharmacokinetics. Drug Des. Devel. Ther..

[6] Texeira M., Pedro M., Nascimento J., Pinto M.M.M., Barbosa C.M. (2019). Development and characterization of PLGA nanoparticles containing 1,3-dihydroxy-2- methylxanthone with improved antitumor activity on a human breast cancer cell line. Pharm. Dev. Technol..

[7] Simeonova S., Georgiev P., Exner K.S., Mihaylov L., Nihtianova D., Koynov K., Balashev K. (2018). Kinetic study of gold nanoparticles synthesized in the presence of chitosan and citric acid. Colloids Surf. A..

[8] Montalbán M.G., Coburn J., Lozano-Pérez A.A., Cenis J.L., Víllora G., Kaplan D. (2018). Production of curcumin-loaded silk fibroin nanoparticles for cancer therapy. Nanomaterials.

[9] Crivelli B., Bari E., Perteghella S., Catenacci L., Sorrenti M., Mocchi M., Faragò S., Tripodo G., Prina-Mello A., Torre M.L. (2019). Silk fibroin nanoparticles for celecoxib and curcumin delivery: ROS-scavenging and anti-inflammatory activities in an in vitro model of osteoarthritis. Eur. J. Pharm. Biopharm..

[10] Krishnakumar N., Sulfikkarali N., RajendraPrasad N., Karthikeyan S. (2011). Enhanced anticancer activity of naringenin-loaded nanoparticles in human cervical (HeLa) cancer cells. Biomed. Prev. Nutr..

[11] Wen J., Liu B., Yuan E., Ma Y., Zhu Y. (2010). Preparation and physicochemical properties of the complex of naringenin with hydroxypropyl-β-cyclodextrin. Molecules.

[12] Semalty A., Semalty M., Singh D., Rawat M.S.M. (2010). Preparation and characterization of phospholipid complexes of naringenin for effective drug delivery. J. Incl. Phenom. Macrocycl. Chem..

[13] Aznar-Cervantes S.D., Lozano-Pérez A.A., García Montalbán M.G., Víllora G., Vicente-Cervantes D., Cenis J.L. (2015). Importance of refrigeration time in the electrospinning of silk fibroin aqueous solutions. J. Mater. Sci..

[14] Vepari C., Kaplan D.L. (2007). Silk as biomaterial. Prog. Polym. Sci..

[15] Belhaj Khalifa I., Ladhari N., Touay M. (2012). Application of sericin to modify textile supports. J. Text. Inst..

[16] Mita K., Ichimura S., James T.C. (1994). Highly repetitive structure and its organization of the silk fibroin gene. J. Mol. Evol..

[17] MacIntosh A.C., Kearns V.R., Crawford A., Hatton P.V. (2008). Skeletal tissue engineering using silk biomaterials. J. Tissue Eng. Regener. Med..

[18] Pritchard E.M., Dennis P.B., Omenetto F., Naik R.R., Kaplan D.L. (2012). Physical and chemical aspects of stabilization of compounds in silk. Biopolymers.

[19] Montalbán M.G., Chakraborty S., Peña-García J., Verli H., Villora G., Pérez-Sánchez H., Díaz-Baños F.G. (2020). Molecular insight into silk fibroin based delivery vehicle for amphiphilic drugs: Synthesis, characterization and molecular dynamics studies. J. Mol. Liq..

[20] Wongpinyochit T., Uhlmann P., Urquhart A.J., Seib F.P. (2015). PEGylated silk nanoparticles for anticancer drug delivery. Biomacromolecules.

[21] Mottaghitalab F., Kiani M., Farokhi M., Kundu S.C., Reis R.L., Gholami M., Bardania H., Dinarvand R., Geramifar P., Beiki D. (2017). Targeted delivery system based on gemcitabine-loaded silk fibroin nanoparticles for lung cancer therapy. ACS Appl. Mater. Interfaces.

[22] Asakura T., Watanabe Y., Uchida A., Minagawa H. (1984). NMR of silk fibroin. Carbon-13 NMR study of the chain dynamics and solution structure of Bombyx mori silk fibroin. Macromolecules.

[23] Ajisawa A. (1997). Dissolution aqueous of silk fibroin with calciumchloride / ethanol solution. J. Sericultural Sci. Japan.

[24] Wang Y., Rudym D.D., Walsh A., Abrahamsen L., Kim H.-J., Kim H.S., Kirker-Head C., Kaplan D.L. (2008). In vivo degradation of three-dimensional silk fibroin scaffolds. Biomaterials.

[25] Phillips D.M., Drummy L.F., Conrady D.G., Fox D.M., Naik R.R., Stone M.O., Trulove P.C., De Long H.C., Mantz R.A. (2004). Dissolution and regeneration of bombyx mori silk fibroin using ionic liquids. J. Am. Chem. Soc..

[26] Carissimi G., Lozano-Pérez A.A., Montalbán M.G., Aznar-Cervantes S.D., Cenis J.L., Víllora G. (2019). Revealing the influence of the degumming process in the properties of silk fibroin nanoparticles. Polymers.

[27] Lozano-Pérez A.A., Montalbán M.G., Aznar-Cervantes S.D., Cragnolini F., Cenis J.L., Víllora G. (2015). Production of silk fibroin nanoparticles using ionic liquids and high-power ultrasounds. J. Appl. Polym. Sci..

[28] Blois M.S. (1958). Antioxidant determinations by the use of a stable free radical. Nature.

[29] Ak T., Gülçin I. (2008). Antioxidant and radical scavenging properties of curcumin. Chem. Biol. Interact..

[30] Qiao F., Zhang J., Wang J., Du B., Huang X., Pang L., Zhou Z. (2017). Silk fibroin-coated PLGA dimpled microspheres for retarded release of simvastatin. Colloids Surf. B.

[31] Subia B., Kundu S.C. (2013). Drug loading and release on tumor cells using silk fibroin-albumin nanoparticles as carriers. Nanotechnology.

[32] Zhang Y.-Q., Shen W.-D., Xiang R.-L., Zhuge L.-J., Gao W.-J., Wang W.-B. (2007). Formation of silk fibroin nanoparticles in water-miscible organic solvent and their characterization. J. Nanopart. Res..

[33] Zhao Z., Xie M., Li Y., Chen A., Li G., Zhang J., Hu H., Wang X., Li S. (2015). Formation of curcumin nanoparticles via solution-enhanced dispersion by supercritical CO_2_. Int. J. Nanomed..

[34] Lozano-Pérez A.A., Gil A.L., Pérez S.A., Cutillas N., Meyer H., Pedreño M., Aznar-Cervantes S.D., Janiak C., Cenis J.L., Ruiz J. (2015). Antitumor properties of platinum(IV) prodrug-loaded silk fibroin nanoparticles. Dalton Trans..

[35] Barth A., Zscherp C. (2002). What vibrations tell us about proteins. Q. Rev. Biophys..

[36] Venyaminov S.Y., Kalnin N.N. (1990). Quantitative IR spectrophotometry of peptide compounds in water (H_2_O) solutions. II. Amide absorption bands of polypeptides and fibrous proteins in alpha-, beta-, and random coil conformations. Biopolymers.

[37] Machado N.F., Batista de Carvalho L.A., Otero J.C., Marques M.P. (2013). A conformational study of hydroxyflavones by vibrational spectroscopy coupled to DFT calculations. Spectrochim. Acta, Part A..

[38] Unsalan O., Erdogdu Y., Gulluoglu M.T. (2009). FT-Raman and FT-IR spectral and quantum chemical studies on some flavonoid derivatives: Baicalein and Naringenin. J. Raman Spectrosc..

[39] Lozano-Pérez A.A., Rivero H.C., Pérez Hernández M.d.C., Pagán A., Montalbán M.G., Víllora G., Cénis J.L. (2017). Silk fibroin nanoparticles: Efficient vehicles for the natural antioxidant quercetin. Int. J. Pharm..

[40] Pérez S.A., Montalbán M.G., Carissimi G., Licence P., Víllora G. (2020). In vitro cytotoxicity assessment of monocationic and dicationic pyridinium-based ionic liquids on HeLa, MCF-7, BGM and EA.hy926 cell lines. J. Hazard. Mater..

[41] Md S., Gan S.Y., Haw Y.H., Ho C.L., Wong S., Choudhury H. (2018). In vitro neuroprotective effects of naringenin nanoemulsion against β-amyloid toxicity through the regulation of amyloidogenesis and tau phosphorylation. Int. J. Biol. Macromol..

[42] Stompor M., Uram Ł., Podgórski R. (2017). In Vitro Effect of 8-Prenylnaringenin and Naringenin on Fibroblasts and Glioblastoma Cells-Cellular Accumulation and Cytotoxicity. Molecules.

[43] Jabbari M., Jabbari A. (2016). Antioxidant potential and DPPH radical scavenging kinetics of water-insoluble flavonoid naringenin in aqueous solution of micelles. Colloids Surf. A.

[44] De Martino L., Mencherini T., Mancini E., Aquino R.P., De Almeida L.F.R., De Feo V. (2012). In vitro phytotoxicity and antioxidant activity of selected flavonoids. Int. J. Mol. Sci..

[45] Peppas N.A., Narasimhan B. (2014). Mathematical models in drug delivery: How modeling has shaped the way we design new drug delivery systems. J. Controlled Release.

[46] Siepmann J., Peppas N.A. (2011). Higuchi equation: Derivation, applications, use and misuse. Int. J. Pharm..

